# Characterization, frequencies and comparison of child abuse reporting rates: a comparative study, Brazil, 2011-2021

**DOI:** 10.1590/S2237-96222025v34e20240545.en

**Published:** 2025-10-10

**Authors:** João Victor de Paula Correia, Fernando Silva-Oliveira, Raquel Conceição Ferreira, Patrícia Maria Pereira de Araújo Zarzar

**Affiliations:** 1 Universidade Federal de Minas Gerais, Departamento de Saúde Bucal da Criança e do Adolescente, Belo Horizonte, MG, Brazil; 2 Universidade Federal de Minas Gerais, Departamento de Odontologia Social e Preventiva, Belo Horizonte, MG, Brazil

**Keywords:** Child Abuse, Domestic Violence, Mandatory Reporting, Health Information Systems, Comparative Study, Maltrato a los Niños, Violencia Doméstica, Notificación Obligatoria, Sistemas de Información en Salud, Estudio Comparativo

## Abstract

**Objectives:**

To describe reported cases of child abuse that occurred from 2011 to 2021 in Brazil and to compare the annual reporting rates of physical, psychological and sexual violence, and neglect/abandonment by sex and age group.

**Methods:**

Data on all cases of violence against children reported on the Notifiable Health Conditions Information System each year were analyzed. The proportions of reported cases for each type of violence were calculated according to age group (0-4 years and 5-9 years) and sex. The cases were characterized according to occurrence, victim and perpetrator, also stratified by age group and sex. Annual reporting rates (per 100,000) were obtained by sex and age group. Comparison of occurrence by sex and age group was made by obtaining the ratio between rates each year.

**Results:**

Most of the 401,058 reports identified were of victims aged 0-4 years (63.5%), cases occurring at home (84.9%) and with the mother (52.5%) and the father (34.6%) as the most frequent perpetrators. Throughout the period, the highest reporting rates were for neglect/abandonment of children of both sexes aged 0-4 years. Psychological and sexual violence were approximately two and 3.5 times, respectively, more frequently reported among girls in both age groups.

**Conclusions:**

Neglect/abandonment is the type of violence most reported by health professionals in the period studied and most frequently victimizes younger children, regardless of sex. Girls suffer sexual and psychological violence more than boys.

## Resumen

**Objetivos:**

Describir los casos de maltrato infantil notificados entre 2011 y 2021 en Brasil y comparar las tasas anuales de notificación de violencia física, psicológica, sexual y negligencia/abandono por sexo y grupo de edad.

**Métodos:**

Se analizaron los datos de todos los casos de violencia contra niños notificados anualmente al Sistema de Información de Enfermedades de Notificación Obligatoria. Se calcularon las proporciones de casos notificados para cada tipo de violencia según el grupo de edad (0-4 años y 5-9 años) y el sexo. Los casos se caracterizaron según la ocurrencia, la víctima y el agresor, y se estratificaron por grupo de edad y sexo. Las tasas anuales de notificación (por 100.000) se obtuvieron por sexo y grupo de edad. La comparación de la ocurrencia por sexo y grupo de edad se realizó mediante la razón entre las tasas de cada año.

**Resultados:**

La mayoría de las 401.058 notificaciones identificadas fueron de víctimas de 0 a 4 años (63,5%), de casos ocurridos en residencias (84,9%) y con la madre (52,5%) y el padre (34,6%) como los agresores más frecuentes. A lo largo del período, las tasas de notificación más altas fueron de negligencia/abandono en niños de 0 a 4 años, en ambos sexos. La violencia psicológica y sexual fueron aproximadamente dos y 3,5 veces, respectivamente, más frecuentes entre las niñas en ambos grupos de edad.

**Conclusione:**

s: La negligencia/abandono es el tipo de violencia más notificado por los profesionales de la salud en el período estudiado y victimiza con mayor frecuencia a niños más pequeños, independientemente del sexo. Las niñas sufren violencia sexual y psicológica más que los niños.

**Ethical aspects**

This research used public domain anonymized databases.

## Introduction

Violence is defined as any use of physical force or power, whether actual or threatened, against oneself or another person, group or community, that has the potential to result in injury, death, psychological harm, deprivation or impaired development and maturation (1).

It is estimated that violence victimizes at least 1 billion children and adolescents aged 2 to 17 years every year worldwide (2). The global economic impacts and costs resulting from the consequences of violence against children can reach seven trillion dollars annually (3). These forms of violence have long-term consequences, increasing the risk of injury, sexually transmitted infections and mental, reproductive and non-communicable disease health problems (4), and are important causes of death in childhood and adulthood (2,5).

Research indicates that the home is the most common place where violence occurs, with perpetrators being people close to the child, usually someone from the child’s own family (6-8). Sociodemographic factors are associated with the occurrence of child abuse, such as race/skin color, age and socioeconomic status; however, these patterns may vary according to the region of the country and time (7,8).

Reporting is one of the most important tools for combating child abuse. The World Health Organization (WHO) recommends that member states strengthen surveillance systems to identify and describe epidemiological behavior in a continuous and timely manner, monitor trends, and identify risk factors. This supports the recommendation and implementation of measures to prevent and respond to violence, as well as the assessment of the impact of multisectoral and networked interventions (9). Evidence indicates that capacity to address and prevent child abuse will be limited, if there is no intelligent information infrastructure that enables continuous and routine monitoring of results (10).

In Brazil, since 2011, all confirmed or suspected cases of domestic, sexual and other types of violence have been subject to mandatory reporting in all public and private health services through the Notifiable Health Conditions Information System (*Sistema de Informação de Agravos de Notificação* - SINAN). Analysis of data reported by SINAN contributes to understanding child abuse in the country by characterizing cases, victims and perpetrators, considering temporal and spatial variations, and enables planning and implementation of effective prevention measures (11). These analyses also make it possible to understand the care provided for this form of harm within the Brazilian National Health System, in addition to highlighting possible gaps in this reporting and information system (12).

Some studies have analyzed data on child abuse reported via SINAN, but they focus mainly on state or municipal data (6,13,14), while those using national data are less common (15). Although these studies identify a significant number of cases of child abuse reported via SINAN, data comparison is limited due to differences in geographic and temporal scope. The latest Violence and Accident Surveillance System (*Sistema de Vigilância de Violências e Acidentes* - VIVA) report, published in 2017, with data from 2013-2014, presented 4,472 cases of child abuse reported in Brazil. However, this report focused on cases treated in urgent and emergency sentinel health care services, excluding from its analysis and dissemination thereof reports made by other health care services, including primary care centers (12). 

With the aim of contributing to the epidemiological surveillance of violence and to the strengthening of the national reporting system, this study described the reported cases of child abuse that occurred between 2011 and 2021 in Brazil and compared the annual reporting rates of physical, psychological and sexual violence and neglect by sex and age groups.

## Methods

### Design 

This is an epidemiological, descriptive and comparative study that used publicly available secondary data from the SINAN.

### Data source and extraction 

The data source was SINAN, which is part of the VIVA. Data extraction and processing were carried out from October 2022 to February 2023. The data were accessed through the DATASUS file transfer area, available on the following website: https://datasus.saude.gov.br/transferencia-de-arquivos/. To download the files, we selected the source (SINAN), modality (data), file type (Viol – Domestic, sexual and/or other violence) and years of interest (2011 to 2021). The files were extracted in *dbc format, separately for each year.

### Study population

The eligible population consisted of suspected or confirmed cases of interpersonal violence against children aged 0-9 years, of both sexes, reported between 2011 and 2021. The year 2011 was considered the initial year, since Ordinance No. 104/2011 was implemented that year, which made reporting of interpersonal violence mandatory for any health facility in the country. In order to select children aged 0-9 years, the age variable was calculated based on date of birth. Age was then categorized into two groups: 0-4 years and 5-9 years, following the classification used by the Brazilian Institute of Geography and Statistics (*Instituto Brasileiro de Geografia e Estatística* - IBGE) (16). Cases involving individuals aged 10 years or older were excluded, as were records with missing data for the sex and age group variables.

### Variables

The variables analyzed were selected from the record fields of the SINAN reporting form for cases of domestic, sexual and other interpersonal violence. The outcome of this study was child abuse. A case of violence was defined based on the type of violence recorded on the form as: physical, psychological/moral, neglect/abandonment, sexual, human trafficking, child labor, torture, financial/economic, legal intervention, other. Each type of violence corresponded to a separate variable in the database. Cases of sexual violence were classified according to the type: sexual harassment, rape, child pornography, sexual exploitation or other.

In order to characterize the cases, we analyzed variables related to the occurrence of violence reported (place of occurrence, repeated occurrence and form of aggression), the victim (age, sex, race/skin color, presence of any type of disability) and the perpetrator (number of people involved, relationship between the victim and the perpetrator, suspicion of alcohol use).

### Data processing and analysis 

The *dbc files extracted from SINAN, separated by year, were integrated into a single *csv file. To minimize information bias, case completeness and duplication analysis was performed. The following variables were selected to determine whether a given report was duplicated: municipality of residence, date of birth, age, sex, report date, case recurrence, and type of violence. In the event of simultaneous coincidence of these variables, this defined duplication, and the first report or the most complete one was kept in the database. Missing values that simultaneously coincided were also considered to be duplicated. Completeness considered the existence of valid records for each variable. Percentage completeness was calculated by dividing the number of existing records for a given variable by the total number of child abuse reporting records in the period analyzed.

In the microdatabase, each reported case corresponded to a row, while the variables were organized into columns, including sex and age group. All reports were considered together in a descriptive analysis to obtain the proportion of cases according to type of violence and stratification by sex and by age group.

In order to compare occurrence of violence according to sex and age group, the annual reporting rates per 100,000 children in each group were calculated, considering the cases of neglect/abandonment and physical, sexual and psychological violence that presented the highest proportions in this study. The rates were calculated by dividing the numerator, which corresponded to the total number of cases reported in each year for each sex and for both age groups (0-4 or 5-9 years of age), by the denominator, which corresponded to the total Brazilian population in the respective age groups and respective sex, according to IBGE estimates (16). In order to compare the rates according to sex, we calculated the ratio between the annual rates, dividing the rate observed for a given type of violence in female children by the corresponding rate in male children. The same procedure was adopted to compare the rates between age groups.

Preparation and linkage of the databases (duplicity and completeness analysis) and calculation of the reporting rates were performed using R statistical software (R Foundation). This was done twice, i.e. by two different researchers, independently, with internal verification and validation of the results. Descriptive analysis of the data was then performed using SPSS statistical software version 26 (IBM Corporation) and Stata version 17 (Timberlake). The study was reported considering the items described in the checklist adapted from STROBE for studies with secondary data, namely STandardized Reporting Of Secondary data Analyses (STROSA) (17).

## Results

A total of 421,107 cases were identified, 19,534 of which were excluded because they were duplicated, leaving 401,573 cases. Of this total, 16 cases (0.003%) were excluded due to lack of information on age, and 499 cases (0.12%) due to lack of recording of the child’s sex variable, leaving a total of 401,058 reports. The percentage of child abuse cases reported in children aged 0-4 years was 63.5%, and 36.5% in children aged 5-9 years. Regarding the children’s sex, there was similarity in the proportion between girls (54.3%) and boys (45.7%).

The type of violence with the highest proportion of reports was neglect/abandonment (53.5%), being more frequent among boys in both age groups, 0-4 years (53.6%) and 5-9 years (55.4%). Sexual violence was the second most frequent type (28.8%), with a higher proportion among girls in both age groups, 0-4 years (78.6%) and 5-9 years (73.3%), with rape having the highest proportion of reports. Physical violence was the third type with the highest proportion (28.2%), with similar values among girls and boys in both age groups (Table 1).

**Table 1:** Distribution of the number and proportion of reported cases of child abuse by type of violence. Brazil, 2011-2021 (n=401,058)

Type of violence (n and % completeness) a	Child age groups and sex				Total cases n (%)
	0-4 years		5-9 years		
	Female	Male	Female	Male	
	n (%)	n (%)	n (%)	n (%)	
Neglect/abandonment (n=382,501; 95.4%)					
Yes	72,640 (46.4)	83,782 (53.6)	21,446 (44.6)	26,599 (55.4)	204,467 (53.5)
No	56,884 (65.0)	30,649 (35.0)	55,466 (61.3)	35,035 (38.7)	178,034 (46.5)
Sexual (n=372,448; 92.9%)					
Yes	37,481 (78.6)	10,222 (21.4)	43,649 (73.3)	15,898 (26.7)	107,250 (28.8)
**Types of sexual violence** ^b^					
Rape (n=104,240; 97.2%)^c^	21,427 (77.8)	6,125 (22.2)	27,648 (72.7)	10,382 (27.3)	65,582 (62.9)
Harassment (n=103,285; 96.3%)^c^	10,023 (79.3)	2,617 (20.7)	14,959 (76.6)	4,570 (23.4)	32,169 (31.2)
Sexual exploitation (n=102,193; 95.3%)^c^	925 (78.3)	257 (21.7)	1,311 (72.0)	510 (28.0)	3,003 (2.9)
Child pornography (n=102,286; 95.4%)^c^	905 (73.5)	326 (26.5)	1,626 (69.4)	716 (30.6)	3,573 (3.5)
Other (n=100,454; 93.7%)^c^	4,022 (80.8)	953 (19.2)	3,241 (75.5)	1,053 (24.5)	9,269 (9.2)
Type of sexual violence not recorded	5,679 (78.8)	1,529 (21.2)	4,134 (70.1)	1,765 (29.9)	13,107 (12.2)
No	88,730 (47.5)	98.083 (52.5)	33,933 (43.3)	44,452 (56.7)	265,198 (71.2)
Physical (n=375,447; 93.6%)					
Yes	31,122 (52.8)	27,775 (47.2)	22,591 (47.9)	24,571 (52.1)	106,059 (28.2)
No	95,479 (53.5)	82,963 (46.5)	54,153 (59.5)	36,793 (40.5)	269,388 (71.8)
Psychological/moral (n=367,712; 91.7%)					
Yes	16,381 (62.5)	9,843 (37.5)	21,579 (59.5)	14,662 (40.5)	62,465 (17.0)
No	107,830 (52.4)	97,959 (47.6)	54,297 (54.6)	45,161 (45.4)	305,247 (83.0)
Torture (n=365,481; 91.1%)					
Yes	1,651 (57.7)	1,211 (42.3)	1,882 (58.4)	1,342 (41.6)	6,086 (1.7)
No	121,951 (53.4)	106,254 (46.6)	73,230 (55.8)	57,960 (44.2)	359,395 (98.3)
Financial/economic (n=367,338; 91.6%)					
Yes	784 (55.4)	630 (44.6)	464 (53.0)	411 (47.0)	2,289 (0.6)
No	123.476 (53.5)	107,242 (46.5)	75,111 (55.9)	59,220 (44.1)	365,049 (99.4)
**Child labor** (n=367,347; 91.6%)					
Yes	320 (48.4)	341 (51.6)	614 (46.8)	697 (53.2)	1,972 (0.5)
No	123.996 (53.6)	107,543 (46.4)	74,919 (56.0)	58,917 (44.0)	365,375 (99.5)
**Legal intervention** (n=367,214; 91.6%)					
Yes	205 (49.6)	208 (50.4)	198 (56.9)	150 (43.1)	761 (0.2)
No	123.976 (53.5)	107,639 (46.5)	75,340 (55.9)	59,498 (44.1)	366,453 (99.8)
**Human trafficking** (n=367,591; 91.6%)					
Yes	88 (63.3)	51 (36.7)	54 (56.8)	41 (43.2)	234 (0.1)
No	124.260 (53.5)	107,849 (46.5)	75,617 (55.9)	59,631 (44.1)	367,357 (99.9)
Other (n=363,515; 90.6%)					
Yes	4,629 (54.0)	3,941 (46.0)	1,834 (52.2)	1,680 (47.8)	12,084 (3.3)
No	118,275 (53.4)	103,263 (46.6)	72,530 (55.8)	57,363 (44.2)	351,431 (96.7)

^a^% completeness: Percentage completeness was calculated by dividing the number of existing records for a given variable by the total number of child abuse report records in the period analyzed; ^b^One or more types of violence may have been recorded for the same case of sexual violence, as such, the sum of the types of sexual violence will not result in the total records of occurrence of sexual violence; ^c^There was loss of data in the recording of type of sexual violence and, for each type of sexual violence, there is a record total, as such, the number of cases for each type, separately, does not coincide with the total number of records of sexual violence.

The place of occurrence with the highest proportion of among the reported cases was the child’s home (74.8%). Bodily force/beating was the most frequent form of aggression (22.2%). The proportion of repeat cases was 40.4%, being more frequent in girls. The highest proportion of reports was for children of mixed race/skin color (46.2%), involving one perpetrator (66.8%). Mothers were the perpetrators in more than half of the reported cases (52.5%), followed by fathers (34.6%). In 18.2% of the cases, there was a record of suspected alcohol use by the perpetrator (Table 2).

**Table 2:** Distribution of the number and proportion of reported cases of child abuse by race/skin color of the victim, number perpetrators involved, relationship between the perpetrator and the victim and suspected use of alcohol by the perpetrator. Brazil, 2011-2021 (n=401,058)

Variables (n and % completeness)^a^	Child age groups and sex					
	0-4 years		5-9 years			
	Female	Male	Female	Male		
Characterization of occurrence	n (%)	n (%)	n (%)	n (%)		Total n (%)
**Place of occurrence** (n=351,282; 87.6%)						
Home	89,298 (54.2)	75,436 (45.8)	58,086 (59.1)	40,130 (40.9)		262,950 (74.8)
Public thoroughfare	5,161 (45.4)	6,213 (54.6)	3,977 (39.9)	5,991 (60.1)		21,342 (6.1)
School	2,840 (59.6)	1,924 (40.4)	2,456 (39.3)	3,792 (60.7)		11,012 (3.1)
Commerce/services	3,269 (47.2)	3,657 (52.8)	881 (49.3)	905 (50.7)		8,712 (2.5)
Outros (Collective housing + Bar or similar + Sports facility + Industries and construction + others)	16,432 (50.7)	15,997 (49.3)	7,721 (52.0)	7,116 (48.0)		47,266 (13.5)
**Repeated occurrence** (n=245,932; 61.3%)						
Yes	26,622 (57.7)	19,482 (42.3)	31,627 (59.4)	21,593 (40.6)		99,324 (40.4)
No	51,320 (51.6)	48,097 (48.4)	25,008 (53.0)	22,183 (47.0)		146,608 (59.6)
Form of aggression						
**Bodily force** (n=355,394; 88.6%)						
Yes	22,749 (58.7)	16,092 (41.3)	21,254 (52.9)	18,896 (47.1)		78,991 (22.2)
No	96,671 (52.0)	89,333 (48.0)	51,000 (56.4)	39,399 (43.6)		276,403 (77.8)
Threat (n=351,316; 87.6%)						
Yes	7,443 (66.7)	3,722 (33.3)	14,710 (65.7)	7,692 (34.3)		33,567 (9.6)
No	110,778 (52.4)	100,794 (47.6)	56,602 (53.3)	49,575 (46.7)		317,749 (90.4)
Intoxication (n=354,668; 88.4%)						
Yes	7,267 (50.1)	7,224 (49.9)	1,569 (53.0)	1,389 (47.0)		17,449 (4.9)
No	112,241 (53.4)	98,020 (46.6)	70,678 (55.7)	56,280 (44.3)		337,219 (95.1)
Substance/object (n=355,133; 88.5%)						
Yes	4,496 (41.8)	6,250 (58.2)	1,146 (41.9)	1,590 (58.1)		13,482 (3.8)
No	115,059 (53.7)	99,123 (46.3)	71,191 (55.8)	56,278 (44.2)		341,651 (96.2)
Blunt object (n=353,598; 88.2%)						
Yes	1,964 (48.3)	2,099 (51.7)	1,647 (41.3)	2,342 (58.7)		8,052 (2.3)
No	117,000 (53.2)	102,798 (46.8)	70,450 (56.0)	55,298 (44.0)		345,546 (97.7)
Sharp object (n=354,250; 88.3%)						
Yes	2,058 (47.0)	2,325 (53.0)	1,231 (42.4)	1,669 (57.6)		7,283 (2.1)
No	117,208 (53.3)	102,814 (46.7)	70,917 (55.9)	56,028 (44.1)		346,967 (97.9)
Firearm (n=354,503; 88.4%)						
Yes	761 (40.0)	1,143 (60.0)	702 (46.2)	817 (53.8)		3,423 (1.0)
No	118,608 (53.3)	104,038 (46.7)	71,520 (55.7)	56,914 (44.3)		351,080 (99.0)
Hanging (n=354,033; 88.3%)						
Yes	850 (59.6)	576 (40.4)	557 (42.7)	747 (57.3)		2,730 (0.8)
No	118,408 (53.1)	104,431 (46.9)	71,596 (55.7)	56,868 (44.3)		351,303 (99.2)
Other (n=348,980; 87.0%)						
Yes	42,842 (49.5)	43,641 (50.5)	15,865 (51.5)	14,967 (48.5)		117,315 (33.6)
No	74,619 (54.9)	61,240 (45.1)	54,014 (56.4)	41,792 (43.6)		231,665 (66.4)
Characterization of the victim	n (%)	n (%)	n (%)	n (%)		Total n (%)
**Race/skin color** (n=343,034; 85.5%)						
White	54,903 (54.5)	45,856 (45.5)	30,821 (55.6)		24,573 (44.4)	156,153 (45.5)
Black	7,088 (54.9)	5,825 (45.1)	5,784 (56.5)		4,447 (43.5)	23,144 (6.8)
Asian	594 (57.7)	435 (42.3)	463 (57.7)		340 (42.3)	1,832 (0.5)
Mixed race	51,924 (52.3)	47,370 (47.7)	33,500 (56.5)		25,753 (43.5)	158,547 (46.2)
Indigenous	1,419 (68.4)	655 (31.6)	950 (74.0)		334 (26.0)	3,358 (1.0)
**Any type of disability/disorder** (n=302,528; 75.4%)						
Yes	2,931 (51.9)	2,719 (48.1)	3,094 (42.6)		4,165 (57.4)	12,909 (4.3)
No	98,483 (54.6)	81,946 (45.4)	63,215 (57.9)		45,975 (42.1)	289,619 (95.7)
Characterization of the perpetrator						
**Number involved** (n=353,325; 88.1%)						
One	78,582 (55.9)	62,069 (44.1)	56,606 (59.4)		38,697 (40.6)	235,954 (66.8)
Two or more	38,502 (48.0)	41,710 (52.0)	17,253 (46.4)		19,906 (53.6)	117,371 (33.2)
Relationship between victim and perpetrator						
Mother (n=360,240; 89.8%)						
Yes	65,876 (46.8)	74,889 (53.2)	21,844 (45.2)		26,467 (54.8)	189,076 (52.5)
No	54,822 (63.1)	32,116 (36.9)	51,912 (61.6)		32,314 (38.4)	171,164 (47.5)
Father (n=351,976; 87.8%)						
Yes	41,856 (49.9)	41,994 (50.1)	18,819 (49.4)		19,257 (50.6)	121,926 (34.6)
No	75,751 (55.2)	61,527 (44.8)	54,209 (58.4)		38,563 (41.6)	230,050 (65.4)
Friends/acquaintances (n=348,570; 86.9%)						
Yes	9,309 (64.8)	5,054 (35.2)	13,858 (57.9)		10,094 (42.1)	38,315 (11.0)
No	107,113 (52.5)	96,946 (47.5)	58,907 (55.5)		47,289 (44.5)	310,255 (89.0)
Stepfather (n=347,474; 86.6%)						
Yes	5,171 (65.6)	2,708 (34.4)	9,137 (71.4)		3,657 (28.6)	20,673 (5.9)
No	111,087 (52.9)	98,878 (47.1)	63,441 (54.3)		53,395 (45.7)	326,801 (94.1)
Stranger (n=348,176; 86.8%)						
Yes	4,236 (60.3)	2,789 (39.7)	3,345 (55.9)		2,641 (44.1)	13,011 (3.7)
No	112,172 (53.0)	99,363 (47.0)	69,110 (55.9)		54,520 (44.1)	335,165 (96.3)
Sibling (n=348,305; 86.9%)						
Yes	2,251 (59.5)	1,534 (40.5)	2,944 (59.0)		2,050 (41.0)	8,779 (2.5)
No	114,337 (53.2)	100,493 (46.8)	69,611 (55.8)		55,085 (44.2)	339,526 (97.5)
Caregiver (n=347,383; 86.6%)						
Yes	2,134 (59.4)	1,460 (40.6)	839 (55.2)		682 (44.8)	5,115 (1.5)
No	114,049 (53.2)	100,316 (46.8)	71,544 (55.9)		56,359 (44.1)	342,268 (98.5)
Stepmother (n=347,617; 86.7%)						
Yes	590 (53.2)	519 (46.8)	878 (52.9)		782 (47.1)	2,769 (0.8)
No	115,769 (53.4)	101,162 (46.6)	71,643 (56.0)		56,274 (44.0)	344,848 (99.2)
**Suspected use of alcohol** (n=217,387; 54.2%)						
Yes	13,019 (57.1)	9,773 (42.9)	10,022 (59.4)		6,845 (40.6)	39,659 (18.2)
No	57,836 (52.3)	52,838 (47.7)	36,174 (53.9)		30,880 (46.1)	177,728 (81.8)

^a^% completeness: Percentage completeness was calculated by dividing the number of existing records for a given variable by the total number of child abuse report records in the period analyzed.

**Figure 1:**
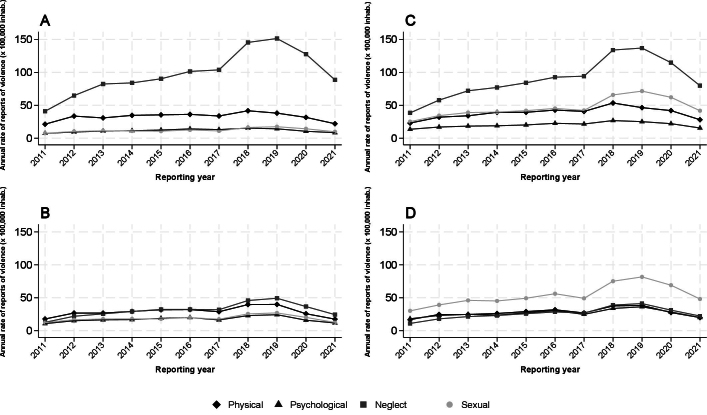
Annual reporting rates (per 100,000 children) of physical, psychological and sexual child abuse and neglect/abandonment among male children aged 0-4 years (A), male children aged 5-9 years (B), female children aged 0-4 years (C) and female children aged 5-9 years (D). Brazil, 2011-2021 (n=401,058)

The highest rates of reported child abuse were for neglect/abandonment in the 0-4 age group for both sexes, with an increase from 2011 to 2019 and a similar pattern of reduction between 2020 and 2021. Rates of sexual violence were the second highest for girls aged 0-4 and the highest for those aged 5-9, with an increase in 2018 and 2019 and a reduction in 2020 and 2021. For boys, the reporting rates of physical, psychological and sexual violence showed little variation over the period. Among girls, the variations in the rates of physical and psychological violence were lower in both age groups and, for girls aged 5-9, the neglect rates were also very similar throughout the period (Figure 1).

The ratios of rates between girls and boys revealed that violence among girls had higher reporting rates for sexual and psychological violence in both age groups for all years. In the case of sexual violence, among children aged 0-4 years, reporting rates were 3.33 times to 4.25 times higher for girls compared to boys. Among girls aged 5-9 years, this ratio varied from 2.35 times to 3.84 times. Reporting rates for psychological violence among girls varied little between ages, being 1.39 time to 2.09 times higher than those for boys. Regarding neglect, rates were approximately 10% lower for girls. In the case of physical violence, rates among girls aged 0-4 years were 8% to 32% higher in the period when compared to rates for boys in the same age group. Among the 5-9 year old group, the rates of physical violence between boys and girls showed practically no difference, but showed a slight tendency to be lower among girls (Table 3).

**Table 3:** Ratio of the reporting rates of physical, psychological and sexual violence and neglect/abandonment between female victims and male victims aged 0-4 years and 5-9 years. Brazil, 2011-2021 (n=401,058)

Ratio: rates of violence against female children/rates of violence against male children aged 0-4 years				
Year	Physical	Psychological	Neglect	Sexual
2011	1.08	1.85	0.93	3.33
2012	0.95	1.80	0.89	3.36
2013	1.10	1.69	0.87	3.44
2014	1.13	1.66	0.91	3.62
2015	1.10	1.61	0.93	3.90
2016	1.17	1.60	0.91	3.35
2017	1.20	1.64	0.90	3.82
2018	1.28	1.74	0.92	4.04
2019	1.21	1.76	0.90	4.08
2020	1.32	2.09	0.89	4.33
2021	1.27	1.85	0.90	4.25
Ratio: rates of violence against female children/rates of violence against male children aged 5-9 years				
2011	0.94	1.68	0.86	2.45
2012	0.91	1.60	0.81	2.35
2013	0.92	1.50	0.83	2.59
2014	0.89	1.39	0.79	2.51
2015	0.91	1.44	0.80	2.74
2016	0.99	1.52	0.88	2.82
2017	0.93	1.51	0.83	2.82
2018	0.94	1.47	0.84	2.94
2019	0.96	1.49	0.83	3.02
2020	1.07	1.77	0.85	3.41
2021	1.13	1.71	0.91	3.84

When comparing age groups, the highest rates of physical violence and neglect were found among children aged 0-4 years, compared to those aged 5-9 years. This difference was more pronounced for neglect, the rates of which were more than 2.8 times higher among children aged 0-4 years in all the years studied. On the other hand, the reporting rates of psychological and sexual violence were higher among children aged 5-9 years (Table 4).

**Table 4:** Ratio of the reporting rates of physical, psychological and sexual violence and neglect/abandonment between victims aged 0-4 years and 5-9 years of the female sex and the male sex. Brazil, 2011-2021 (n=401,058)

Ratio: rates of violence 0-4 years/rates of violence 5-9 years for female children				
Year	Physical	Psychological	Neglect	Sexual
2011	1.36	0.75	3.48	0.83
2012	1.29	0.69	3.21	0.87
2013	1.38	0.74	3.36	0.84
2014	1.50	0.80	3.33	0.87
2015	1.35	0.74	3.25	0.84
2016	1.34	0.74	3.25	0.75
2017	1.51	0.87	3.53	0.85
2018	1.42	0.79	3.44	0.87
2019	1.20	0.69	3.32	0.87
2020	1.51	0.77	3.67	0.89
2021	1.40	0.77	3.57	0.86
Ratio: rates of violence 0-4 years/rates of violence 5-9 years for male children				
2011	1.19	0.68	3.21	0.61
2012	1.25	0.62	2.95	0.61
2013	1.15	0.65	3.21	0.63
2014	1.18	0.67	2.87	0.60
2015	1.11	0.66	2.80	0.59
2016	1.12	0.70	3.13	0.63
2017	1.17	0.79	3.25	0.63
2018	1.04	0.66	3.17	0.63
2019	0.95	0.58	3.07	0.64
2020	1.22	0.66	3.48	0.70
2021	1.25	0.71	3.62	0.78

## Discussion

This study shows a high proportion and high reporting rates of abuse in the form of neglect, especially in the 0-4 age group, and sexual violence, especially among girls. Domestic violence is a serious problem, with a greater proportion of cases occurring in the child’s home, with the mother, but also the father, being the main perpetrators.

The limitations of the study lie in the data source. Although the SINAN is an important tool for epidemiological surveillance in Brazil, its data depend on records made by health professionals and health services, and the quality of these data can be compromised by completion errors and omission of reports, which can result in underreporting or reports with important information missing. This situation can be aggravated in areas with less health service coverage or among more vulnerable populations. Another limitation refers to the duplication and completeness of variables, which limits the characterization of occurrences of violence. For example, in this study, it was not possible to use the education level variable due to low completeness. However, non-duplicity was found for 99.9% of the records, and, in general, the variables analyzed presented completeness above 85%, these being values above what is considered acceptable for non-duplicity (>95%) and good completeness (≥75.1%) (18). Some variables showed regular completeness (50.1% to 75.0%): namely suspected alcohol use and repeated occurrence. This low completeness may introduce bias, as analyses based on incomplete data may not represent all reported cases. The external validity of the study is limited, as the data do not represent all reported cases among Brazilian children.

The home as the main place of occurrence (74.8%) is consistent with other studies that analyzed data from SINAN and other sources of data on violence both in Brazil and abroad (6,7,13). However, some local studies have presented lower proportions, ranging from 50% to 60% of violence cases occurring at home (14), which suggests the existence of regional differences and highlights the importance of understanding these variations in order to adapt actions and strategies. The proximity of the perpetrators, often parents, also explains the predominance of the home as the place where violence occurs (6).

Children of mixed race/skin color had the highest proportion of reports. When considering Black children (mixed race and Black together), this proportion reached 53%. National (14) and international (13,19) studies corroborate this trend, also recognized by the WHO (19). However, some Brazilian studies, including regional analyses of SINAN data, indicate a higher proportion of reports among White children (6). Given that Black children represent approximately 56.7% of the Brazilian child population (16), this discrepancy between the proportion of Black children in the national population and in reports of violence may suggest possible cases of underidentification and/or underreporting of violence involving Black children. Another hypothesis is that these children have less access to health services, which could impact identification and reporting. It is also important to reflect on whether, due to structural racism, health professionals may be making cases of violence against Black children invisible. Therefore, for child abuse prevention and control measures to be effective, it is essential that they are complemented by comprehensive policies to combat structural racism.

This study identified a high proportion of perpetrators who were possibly under the influence of alcohol, in line with WHO evidence that children in families or communities with a history of alcohol abuse are more vulnerable to violence (20,21). Policies to regulate alcohol use, especially in the presence of children, are essential. It is recommended that the SINAN reporting form include information on use of other substances as well.

Children aged 0–4 years had the highest rates of reported neglect. This age group is identified as vulnerable to neglect due to their dependence on caregivers and difficulty in establishing protective social contacts. These findings are in line with studies showing that neglect is the most frequent type of child abuse, especially among younger children (6,13,14,22). Studies show that neglect can cause serious harm to children’s development, affecting their cognition, attention, and social behavior. These forms of harm can persist throughout life (22). Universal prevention measures are essential, both at the individual and community levels, and health care plays a key role in early detection of neglect (11,23).

Vulnerable situations, such as lack of access to health services and financial resources, often result in professionals disregarding neglect, a situation that is aggravated in contexts of social inequality. In these cases, it is important to recognize that neglect is not only caused by or the fault of parents, but also a reflection of the structural shortcomings of the State. It is crucial that professionals identify these cases, recognize them as neglect, and report them, even when they are not cases of parental neglect. Differentiating between parental and structural neglect on SINAN could facilitate a more accurate description of the situation of this type of violence, favoring appropriate intervention strategies. Improving the quality of records also involves professional training, as studies indicate that professionals have difficulty identifying and addressing neglect (23,24). Another important point that may influence health professionals’ decision not to report suspected cases is the distorted perception that neglect is a form of abuse with less potential to cause harm (24).

Reported rates of sexual violence in girls were more than double, even reaching more than four times higher than those observed for boys. Findings indicate an increased risk of sexual violence as girls grow older (25). Patriarchal culture and power relations play a crucial role in perpetuating sexual violence against children, reflecting entrenched gender inequalities in society (26,27). Historically, women have been considered socially fragile, submissive to parents and partners, perpetuating a cycle of domination (26). This is reflected in the subordination of children, especially girls, who are at greater risk of physical and sexual violence due to rape culture and patriarchy (26). Combating child sexual abuse involves confronting sexism, misogyny, and gender oppression.

Physical violence was the third most reported type of violence, unlike other studies that reported a higher proportion of physical violence compared to sexual abuse (6.13). This may suggest a possible cultural normalization of physical violence against children in Brazil, evidenced by the acceptance of physical punishment as a disciplinary method, despite legislative initiatives (Law No. 13010/2014) and recent debates on the subject (28,29). This acceptance may extend to health professionals. In addition, there is a normalized belief, including among health professionals, that physical abuse should only be considered when there are visible injuries, which may result in the failure to identify cases in which the child does not present physical signs, as well as resulting in case underreporting (30).

The increase in child abuse reporting rates over the years covered in this study can be attributed to the implementation of Ordinance No. 104/2011, which made child abuse a compulsorily reportable condition at all levels of health care in Brazil. However, from 2020 onwards, a decrease in reporting rates was found, possibly due to the COVID-19 pandemic, which may have led to underreporting. Social isolation, school closures, and health services concentrating on dealing with the pandemic reduced children’s contact with health professionals and made it unlikely that they would establish protective social ties in their daily lives. However, this decrease in reporting should not be interpreted as a reduction in the occurrence of violence, since the incidence of violence increased during this period, corroborating the idea that violence occurs mainly within the domestic environment (19).

In Brazil, there are other channels for reporting child abuse in addition to SINAN, such as the Dial 100 service (*Disque 100*), but the data generated are not integrated into a single system. Monitoring cases would benefit greatly from integration, in line with the guidelines of international organizations, such as the WHO, which highlight the importance of comprehensive and integrated information systems to strengthen surveillance and combat violence. In addition, it is essential to inform health professionals about the mandatory nature of reporting and the use of SINAN as the standard information system, as evidence reveals a lack of awareness (30).

Despite existing policies on combating child abuse in Brazil, there is a clear need for greater investment in other policies to reduce these rates, highlighting the importance of gender policies, respect and protection of females, a culture of peace, combating racism, and the integration of sexual education from an early age. Furthermore, it is crucial to train health and education professionals better in order to deal with the problem. It should be noted that neglect and domestic violence are the main forms of child abuse recorded in Brazil during the period studied, which highlights the crucial role of primary health care. Strengthening surveillance of violence against children, integrating it into primary care, can be an effective measure in combating child abuse.
