# Supplementary material for: Characterization, frequencies and comparison of child abuse reporting rates: a comparative study, Brazil, 2011-2021
Source: Epidemiol Serv Saude. 2025 Oct 10;34:e20240545. doi: 10.1590/S2237-96222025v34e20240545.en (PMC12513467; doi:10.1590/S2237-96222025v34e20240545.en)
Supplement: Translation

# Translation: Caracterização, frequências e comparação das taxas de notificação de violência infantil: estudo comparativo, Brasil, 2011-2021

**DOI:** 10.1590/S2237-96222025v34e20240545.pt

**Authors:** João Victor de Paula Correia, Fernando Silva-Oliveira, Raquel Conceição Ferreira, Patrícia Maria Pereira de Araújo Zarzar

**Affiliations:** 1 Universidade Federal de Minas Gerais, Departamento de Saúde Bucal da Criança e do Adolescente, Belo Horizonte, MG, Brasil; 2 Universidade Federal de Minas Gerais, Departamento de Odontologia Social e Preventiva, Belo Horizonte, MG, Brasil

**Keywords:** Maus-tratos Infantis, Violência Doméstica, Notificação de Abuso, Sistemas de Informação em Saúde, Estudo Comparativo

## Abstract

**Objetivos:**

Descrever os casos notificados de violência infantil ocorridos de 2011 a 2021 no Brasil e comparar as taxas anuais de notificação de violência física, psicológica, sexual e negligência/abandono por sexo e por grupos etários.

**Métodos:**

Foram analisados os dados de todos os casos de violência contra crianças notificados ao Sistema de Informação de Agravos de Notificação a cada ano. As proporções de casos notificados para cada tipo de violência foram calculadas segundo faixa etária (0-4 anos e 5-9 anos) e sexo. Os casos foram caracterizados quanto à ocorrência, à vítima e ao agressor, também estratificados por faixa etária e sexo. Taxas anuais de notificação (por 100 mil) foram obtidas por sexo e por grupo etário. A comparação na ocorrência por sexo e por grupo etário foi realizada pela obtenção da razão entre taxas em cada ano.

**Resultados:**

A maioria das 401.058 notificações identificadas foram de vítimas de 0-4 anos (63,5%), de casos ocorridos em residências (84,9%) e tendo a mãe (52,5%) e o pai (34,6%) como os agressores mais frequentes. Em todo o período, as maiores taxas de notificação foram de negligência/abandono aos 0-4 anos, em ambos os sexos. As violências psicológica e sexual foram, aproximadamente, duas e 3,5 vezes, respectivamente, mais notificadas entre meninas nos dois grupos etários.

**Conclusões:**

A negligência/abandono é o tipo de violência mais notificada pelos profissionais de saúde no período estudado e vitimiza mais frequentemente crianças mais novas, independente do sexo. As meninas sofrem violência sexual e psicológica mais do que os meninos.

**Aspectos éticos**

Esta pesquisa utilizou bancos de dados de domínio público e anonimizados.

## Introdução

A violência é definida como qualquer uso de força física ou de poder, real ou ameaça, contra si ou outra pessoa, um grupo ou uma comunidade, que tenha possibilidade de resultar em lesões, morte, dano psicológico, privação ou deficiência de desenvolvimento e maturação (1).

Estima-se que a violência vitimize pelo menos 1 bilhão de crianças e adolescentes com idade entre 2 e 17 anos a cada ano no mundo (2). Os impactos econômicos globais e os custos resultantes das consequências das violências contra crianças podem atingir sete trilhões de dólares anuais (3). Essas formas de violência têm consequências de longo prazo, aumentando o risco de lesões, infecções sexualmente transmissíveis e problemas de saúde mental, reprodutiva e não transmissíveis (4), tornando-se importantes causas de morte na vida infantil e adulta (2,5).

Pesquisas indicam que o domicílio é o local mais comum de ocorrência da violência, tendo, como agressores, pessoas próximas à criança, geralmente alguém da própria família (6-8). Fatores sociodemográficos estão associados à ocorrência de violência infantil, como a raça/cor da pele, a idade e a condição socioeconômica; porém, esses padrões podem variar de acordo com a região do país e o tempo (7,8). 

A notificação é um dos importantes instrumentos para o enfrentamento da violência infantil. A Organização Mundial da Saúde (OMS) recomenda que os Estados membros fortaleçam os sistemas de vigilância para identificar e descrever o comportamento epidemiológico de forma contínua e oportuna, monitorar tendências e identificar fatores de risco. Isso sustenta a recomendação e a implementação de medidas para prevenção e resposta à violência, bem como a avaliação do impacto de intervenções multissetoriais e em rede (9). Evidências apontam que a capacidade de enfrentamento e de prevenção da violência infantil é limitada, sem uma infraestrutura de informação inteligente que permita monitoramento contínuo e rotineiro dos resultados (10). 

No Brasil, desde 2011, todos os casos confirmados ou suspeitos de violência doméstica, sexual e outras violências tornaram-se de notificação compulsória em todos os serviços de saúde, públicos ou privados por meio do Sistema de Informação de Agravos de Notificação (Sinan). A análise dos dados notificados pelo Sinan contribui para compreender a violência infantil no país a partir da caracterização dos casos, das vítimas e dos agressores, considerando variações temporais e espaciais, e possibilita o planejamento e a implementação de medidas preventivas eficazes (11). Essas análises também possibilitam compreender os atendimentos relacionados a esses agravos no sistema de saúde nacional, além de evidenciar possíveis lacunas nesse sistema de notificação e de informação (12).

Algumas pesquisas analisaram dados de violência infantil notificados ao Sinan, mas concentram-se principalmente em dados estaduais ou municipais (6,13,14), sendo menos comuns aqueles que utilizam dados nacionais (15). Embora esses estudos identifiquem um número significativo de casos de violência infantil notificados ao Sinan, a comparação dos dados é limitada devido a diferenças nas abrangências geográficas e temporais. O último relatório do Sistema de Vigilância de Violências e Acidentes (VIVA), publicado em 2017, com dados de 2013 a 2014, apresentou 4.472 casos de violência infantil notificados no Brasil. Mas esse relatório se concentrou nos casos atendidos em serviços sentinela de urgência e de emergência, excluindo da análise e da divulgação as notificações feitas por outras unidades, inclusive as da atenção primária (12). 

Visando contribuir com a vigilância epidemiológica de violência e com o fortalecimento do sistema nacional de notificação, este estudo descreveu os casos notificados de violência infantil ocorridos de 2011 a 2021 no Brasil e comparou as taxas anuais de notificação de violência física, psicológica, sexual e de negligência por sexo e por grupos etários. 

## Métodos

### Delineamento

Este é um estudo epidemiológico, descritivo e comparativo, que utilizou dados secundários de acesso público disponibilizados pelo Sinan. 

### Fonte e extração de dados

A fonte de dados foi o Sinan, que faz parte do VIVA. A extração e processamento dos dados foram realizados no período de outubro de 2022 a fevereiro de 2023. Os dados foram acessados pela área de transferência de arquivos do DATASUS, disponível pelo website: https://datasus.saude.gov.br/transferencia-de-arquivos/. Para o download dos arquivos, foram selecionados a fonte (Sinan), a modalidade (dados), o tipo de arquivo (Viol – Violência doméstica, sexual e/ou outras violências) e os anos de interesse (2011 a 2021). Os arquivos foram extraídos em formato *dbc, separadamente para cada ano. 

### População do estudo

A população elegível foram os casos, suspeitos ou confirmados, de violência interpessoal infantil, nas idades de 0-9 anos, de ambos os sexos, notificados no período de 2011 a 2021. O ano de 2011 foi considerado inicial, pois houve a implementação da portaria n° 104/2011, que tornou compulsória a notificação de violência interpessoal por qualquer estabelecimento de saúde do país. Para selecionar as crianças com idade entre 0-9 anos, foi calculada a variável idade a partir da data de nascimento. Em seguida, a idade foi categorizada em dois grupos: 0-4 anos e 5-9 anos, seguindo a classificação empregada pelo Instituto Brasileiro de Geografia e Estatística (IBGE) (16). Foram excluídos casos em indivíduos com 10 anos ou mais, além de registros com dados faltantes para as variáveis sexo e faixa etária.

### Variáveis analisadas

As variáveis analisadas foram selecionadas a partir dos campos de registro da ficha de notificação dos casos de violência doméstica, sexual e outras violências interpessoais do Sinan. O desfecho deste estudo foi a violência infantil. O caso de violência foi definido a partir do registro do tipo de violência na ficha em: física, psicológica/moral, negligência/abandono, sexual, tráfico de seres humanos, trabalho infantil, tortura, financeira/econômica, intervenção legal, outros. Cada tipo de violência correspondeu a uma variável separada na base de dados. Os casos de violência sexual foram classificados segundo o tipo: assédio sexual, estupro, pornografia infantil, exploração sexual ou outros. 

Para caracterizar os casos, foram analisadas variáveis referentes à ocorrência (local de ocorrência, reincidência da ocorrência e meio de agressão), à vítima (idade, sexo, raça/cor da pele, presença de algum tipo de deficiência) e ao agressor (número de envolvidos, relação da vítima com o agressor, suspeita de uso de bebidas alcoólicas). 

### Processamento e análise dos dados

Os arquivos *dbc extraídos do Sinan, separados por ano, foram integrados em um único arquivo de formato *csv. Para minimizar o viés de informação, foi realizada a análise de completitude e de duplicidade dos casos. As seguintes variáveis foram selecionadas para determinar se uma dada notificação era duplicada: município de residência, data de nascimento, idade, sexo, data de notificação, recorrência do caso e tipo de violência. Em caso de coincidência simultânea dessas variáveis, definiu-se haver duplicidade, sendo retida na base a primeira notificação ou aquela mais completa. Valores faltantes simultaneamente coincidentes também foram considerados como duplicidade. A completitude considerou a existência de registros válidos para cada variável. O percentual de completitude foi calculado dividindo-se o número de registros existentes para dada variável pelo total de registros de notificações de violência infantil no período analisado.

Na base de microdados, cada caso notificado correspondia a uma linha, enquanto as variáveis eram organizadas em colunas, incluindo sexo e grupo etário. Todas as notificações foram consideradas conjuntamente em uma análise descritiva, para obtenção da proporção de casos segundo o tipo de violência e estratificação por sexo e por grupos etários. 

Para comparar a ocorrência de violência segundo sexo e grupo etário, foram calculadas as taxas anuais de notificação por 100 mil crianças de cada grupo, considerando os casos de negligência/abandono e de violência física, sexual e psicológica que apresentaram as maiores proporções neste estudo. As taxas foram calculadas dividindo-se o numerador, que correspondeu ao total de casos notificados em cada ano para cada sexo e para cada um dos grupos etários de 0-4 anos ou de 5-9 anos de idade, pelo denominador, que correspondeu à população total brasileira nas respectivas faixas etárias e sexo, conforme estimativas do IBGE (16). Para comparar as taxas segundo o sexo, foi calculada a razão entre as taxas anuais, dividindo-se a taxa observada para determinado tipo de violência em crianças do sexo feminino pela taxa correspondente em crianças do sexo masculino. O mesmo procedimento foi adotado para comparar as taxas entre os grupos etários. 

A preparação e a vinculação das bases de dados (análise de duplicidade e completitude) e o cálculo das taxas de notificação foram realizados empregando-se o software estatístico R (R Foundation) em duplicada por dois pesquisadores, de forma independente, com conferência e validação interna dos resultados. Em seguida, a análise descritiva dos dados foi realizada empregando-se os softwares estatísticos SPSS versão 26 (IBM Corporation) e Stata versão 17 (Timberlake). O estudo foi reportado considerando-se os itens descritos no checklist adaptado do Strobe para estudos com dados secundários, STandardized Reporting Of Secondary data Analyses (STROSA) (17).

## Resultados

Um total de 421.107 casos foram identificados, desses, 19.534 casos foram excluídos, pois eram duplicados, permanecendo 401.573 casos. Desse total, 16 casos (0,003%) foram excluídos devido à ausência de informações sobre a idade, e 499 casos (0,12%) por ausência de registro da variável sexo, restando um total de 401.058 notificações. O percentual de casos de violência infantil notificados em crianças de 0-4 anos foi de 63,5%, e de 36,5% em crianças de 5-9 anos. Com relação ao sexo, houve semelhança na proporção entre meninas (54,3%) e meninos (45,7%).

O tipo de violência com maior proporção de notificações foi a negligência/abandono (53,5%), sendo mais frequente entre meninos em ambos os grupos etários, de 0-4 anos (53,6%) e de 5-9 anos (55,4%). A violência sexual foi o segundo tipo mais frequente (28,8%), com maior proporção entre meninas nos dois grupos etários, de 0-4 anos (78,6%) e de 5-9 anos (73,3%), tendo o estupro a maior proporção de notificação. A violência física foi o terceiro tipo com maior proporção (28,2%), com valores semelhantes entre meninas e meninos nos dois grupos etários (Tabela 1). 

**Tabela 1:** Distribuição do número e proporção dos casos notificados de violência infantil segundo o tipo de violência. Brasil, 2011 a 2021 (n=401.058)

Tipo de violência (n e % de completude)^a^	Grupos etários e sexo da criança				Total de casos n (%)
	0-4 anos		5-9 anos		
	Feminino	Masculino	Feminino	Masculino	
	n (%)	n (%)	n (%)	n (%)	
Negligência/abandono (n= 382.501; 95,4%)					
Sim	72.640 (46,4)	83.782 (53,6)	21.446 (44,6)	26.599 (55,4)	204.467 (53,5)
Não	56.884 (65,0)	30.649 (35,0)	55.466 (61,3)	35.035 (38,7)	178.034 (46,5)
Sexual (n=372.448; 92,9%)					
Sim	37.481 (78,6)	10.222 (21,4)	43.649 (73,3)	15.898 (26,7)	107.250 (28,8)
**Tipos de violência s^e^xual b**					
Estupro (n=104.240; 97,2%)^c^	21.427 (77,8)	6.125 (22,2)	27.648 (72,7)	10.382 (27,3)	65.582 (62,9)
Assédio (n=103.285; 96,3%)^c^	10.023 (79,3)	2.617 (20,7)	14.959 (76,6)	4.570 (23,4)	32.169 (31,2)
Exploração sexual (n=102.193; 95,3%)^c^	925 (78,3)	257 (21,7)	1.311 (72,0)	510 (28,0)	3.003 (2,9)
Pornografia infantil (n=102.286; 95,4%)^c^	905 (73,5)	326 (26,5)	1.626 (69,4)	716 (30,6)	3.573 (3,5)
Outros (n=100.454; 93,7%)^c^	4.022 (80,8)	953 (19,2)	3.241 (75,5)	1.053 (24,5)	9.269 (9,2)
Sem registro de algum tipo de violência sexual	5.679 (78,8)	1.529 (21,2)	4.134 (70,1)	1.765 (29,9)	13.107 (12,2)
Não	88.730 (47,5)	98.083 (52,5)	33.933 (43,3)	44.452 (56,7)	265.198 (71,2)
Física (n=375.447; 93,6%)					
Sim	31.122 (52,8)	27.775 (47,2)	22.591 (47,9)	24.571 (52,1)	106.059 (28,2)
Não	95.479 (53,5)	82.963 (46,5)	54.153 (59,5)	36.793 (40,5)	269.388 (71,8)
Psicológica/moral (n=367.712; 91,7%)					
Sim	16.381 (62,5)	9.843 (37,5)	21.579 (59,5)	14.662 (40,5)	62.465 (17,0)
Não	107.830 (52,4)	97.959 (47,6)	54.297 (54,6)	45.161 (45,4)	305.247 (83,0)
Tortura (n=365.481; 91,1%)					
Sim	1.651 (57,7)	1.211 (42,3)	1.882 (58,4)	1.342 (41,6)	6.086 (1,7)
Não	121.951 (53,4)	106.254 (46,6)	73.230 (55,8)	57.960 (44,2)	359.395 (98,3)
Financeira/econômica (n=367.338; 91,6%)					
Sim	784 (55,4)	630 (44,6)	464 (53,0)	411 (47,0)	2.289 (0,6)
Não	123.476 (53,5)	107,242 (46,5)	75.111 (55,9)	59.220 (44,1)	365.049 (99,4)
**Trabalho infantil** (n=367.347; 91,6%)					
Sim	320 (48,4)	341 (51,6)	614 (46,8)	697 (53,2)	1.972 (0,5)
Não	123.996 (53,6)	107.543 (46,4)	74.919 (56,0)	58.917 (44,0)	365.375 (99,5)
**Intervenção legal** (n=367.214; 91,6%)					
Sim	205 (49,6)	208 (50,4)	198 (56,9)	150 (43,1)	761 (0,2)
Não	123.976 (53,5)	107.639 (46,5)	75.340 (55,9)	59.498 (44,1)	366.453 (99,8)
**Tráfico de seres humanos** (n=367.591; 91,6%)					
Sim	88 (63,3)	51 (36,7)	54 (56,8)	41 (43,2)	234 (0,1)
Não	124.260 (53,5)	107.849 (46,5)	75.617 (55,9)	59.631 (44,1)	367.357 (99,9)
Outros (n=363.515; 90,6%)					
Sim	4.629 (54,0)	3.941 (46,0)	1.834 (52,2)	1.680 (47,8)	12.084 (3,3)
Não	118.275 (53,4)	103.263 (46,6)	72.530 (55,8)	57.363 (44,2)	351.431 (96,7)

^a^% de completude: O percentual de completude foi calculado dividindo-se o número de registros existentes para dada variável pelo total de registros de notificações de violência infantil no período analisado; ^b^Um ou mais tipos de violência podem ser registrados para um mesmo caso de violência sexual, portanto, a soma dos tipos de violência sexual não resultará no total de registros de ocorrência de violência sexual; ^c^Houve dados perdidos no registro do tipo de violência sexual e, para cada tipo de violência sexual, há um total de registro, por isso, o número de casos para cada tipo, separadamente, não é coincidente com o número total de registros de violência sexual.

A maior proporção de local de ocorrência dentre os casos notificados foi a residência da criança (74,8%). A força corporal/espancamento foi o meio de agressão mais frequente (22,2%). A proporção de casos reincidentes foi 40,4%, sendo mais frequente nas meninas. A maior proporção de notificações foi de crianças pardas (46,2%), envolvendo um agressor (66,8%). As mães foram as agressoras em mais da metade dos casos notificados (52,5%), seguidas pelos pais (34,6%). Em 18,2% dos casos houve registro de suspeita de uso de álcool pelo agressor (Tabela 2). 

**Tabela 2:** Distribuição do número e proporção dos casos notificados de violência infantil segundo raça/cor da pele da vítima, número de agressores envolvidos, relação do agressor com a vítima e suspeita de uso de álcool pelo agressor. Brasil, 2011 a 2021 (n=401.058)

Variáveis (n e % de completude)^a^	Grupos etários e sexo da criança					
	0-4 anos		5-9 anos			
	Feminino	Masculino	Feminino	Masculino		
Caracterização da ocorrência	n (%)	n (%)	n (%)	n (%)		Total n (%)
**Local da ocorrência** (n=351.282; 87,6%)						
Residência	89.298 (54,2)	75.436 (45,8)	58.086 (59,1)	40.130 (40,9)		262.950 (74,8)
Via pública	5.161 (45,4)	6.213 (54,6)	3.977 (39,9)	5.991 (60,1)		21.342 (6,1)
Escola	2.840 (59,6)	1.924 (40,4)	2.456 (39,3)	3.792 (60,7)		11.012 (3,1)
Comércio/serviços	3.269 (47,2)	3.657 (52,8)	881 (49,3)	905 (50,7)		8.712 (2,5)
Outros (Habitação coletiva + Bar ou similar + Local de prática esportiva + Indústrias e construção + outros)	16.432 (50,7)	15.997 (49,3)	7.721 (52,0)	7.116 (48,0)		47.266 (13,5)
**Reincidência da ocorrência** (n=245.932; 61,3%)						
Sim	26.622 (57,7)	19.482 (42,3)	31.627 (59,4)	21.593 (40,6)		99.324 (40,4)
Não	51.320 (51,6)	48.097 (48,4)	25.008 (53,0)	22.183 (47,0)		146.608 (59,6)
Meio de agressão						
**Força corporal** (n=355.394; 88,6%)						
Sim	22.749 (58,7)	16.092 (41,3)	21.254 (52,9)	18.896 (47,1)		78.991 (22,2)
Não	96.671 (52,0)	89.333 (48,0)	51.000 (56,4)	39.399 (43,6)		276.403 (77,8)
Ameaça (n=351.316; 87,6%)						
Sim	7.443 (66,7)	3.722 (33,3)	14.710 (65,7)	7.692 (34,3)		33.567 (9,6)
Não	110.778 (52,4)	100.794 (47,6)	56.602 (53,3)	49.575 (46,7)		317.749 (90,4)
Envenenamento (n=354.668; 88,4%)						
Sim	7.267 (50,1)	7.224 (49,9)	1.569 (53,0)	1.389 (47,0)		17.449 (4,9)
Não	112.241 (53,4)	98.020 (46,6)	70.678 (55,7)	56.280 (44,3)		337.219 (95,1)
Substância/objeto (n=355.133; 88,5%)						
Sim	4.496 (41,8)	6.250 (58,2)	1.146 (41,9)	1.590 (58,1)		13.482 (3,8)
Não	115.059 (53,7)	99.123 (46,3)	71.191 (55,8)	56.278 (44,2)		341.651 (96,2)
Objeto contundente (n=353.598; 88,2%)						
Sim	1.964 (48,3)	2.099 (51,7)	1.647 (41,3)	2.342 (58,7)		8.052 (2,3)
Não	117.000 (53,2)	102.798 (46,8)	70.450 (56,0)	55.298 (44,0)		345.546 (97,7)
Objeto perfurocortante (n=354.250; 88,3%)						
Sim	2.058 (47,0)	2.325 (53,0)	1.231 (42,4)	1.669 (57,6)		7.283 (2,1)
Não	117.208 (53,3)	102.814 (46,7)	70.917 (55,9)	56.028 (44,1)		346.967 (97,9)
**Arma de fogo** (n=354.503; 88,4%)						
Sim	761 (40,0)	1.143 (60,0)	702 (46,2)	817 (53,8)		3.423 (1,0)
Não	118.608 (53,3)	104.038 (46,7)	71.520 (55,7)	56.914 (44,3)		351.080 (99,0)
Enforcamento (n=354.033; 88,3%)						
Sim	850 (59,6)	576 (40,4)	557 (42,7)	747 (57,3)		2.730 (0,8)
Não	118.408 (53,1)	104.431 (46,9)	71.596 (55,7)	56.868 (44,3)		351.303 (99,2)
Outros (n=348.980; 87,0%)						
Sim	42.842 (49,5)	43.641 (50,5)	15.865 (51,5)	14.967 (48,5)		117.315 (33,6)
Não	74.619 (54,9)	61.240 (45,1)	54.014 (56,4)	41.792 (43,6)		231.665 (66,4)
Caracterização da vítima	n (%)	n (%)	n (%)		n (%)	Total n (%)
**Raça/cor da pele** (n=343.034; 85,5%)						
Branca	54.903 (54,5)	45.856 (45,5)	30.821 (55,6)		24.573 (44,4)	156.153 (45,5)
Preta	7.088 (54,9)	5.825 (45,1)	5.784 (56,5)		4.447 (43,5)	23.144 (6,8)
Amarela	594 (57,7)	435 (42,3)	463 (57,7)		340 (42,3)	1.832 (0,5)
Parda	51.924 (52,3)	47.370 (47,7)	33.500 (56,5)		25.753 (43,5)	158.547 (46,2)
Indígena	1.419 (68,4)	655 (31,6)	950 (74,0)		334 (26,0)	3.358 (1,0)
**Possui algum tipo de deficiência/transtorno** (n=302.528; 75,4%)						
Sim	2.931 (51,9)	2.719 (48,1)	3.094 (42,6)		4.165 (57,4)	12.909 (4,3)
Não	98.483 (54,6)	81.946 (45,4)	63.215 (57,9)		45.975 (42,1)	289.619 (95,7)
Caracterização do agressor						
**Número de envolvidos** (n=353.325; 88,1%)						
Um	78.582 (55,9)	62.069 (44,1)	56.606 (59,4)		38.697 (40,6)	235.954 (66,8)
Dois ou mais	38.502 (48,0)	41.710 (52,0)	17.253 (46,4)		19.906 (53,6)	117.371 (33,2)
Relação da vítima com o agressor						
Mãe (n=360.240; 89,8%)						
Sim	65.876 (46,8)	74.889 (53,2)	21.844 (45,2)		26.467 (54,8)	189.076 (52,5)
Não	54.822 (63,1)	32.116 (36,9)	51.912 (61,6)		32.314 (38,4)	171.164 (47,5)
Pai (n=351.976; 87,8%)						
Sim	41.856 (49,9)	41.994 (50,1)	18.819 (49,4)		19.257 (50,6)	121.926 (34,6)
Não	75.751 (55,2)	61.527 (44,8)	54.209 (58,4)		38.563 (41,6)	230.050 (65,4)
Amigos/conhecidos (n=348.570; 86,9%)						
Sim	9.309 (64,8)	5.054 (35,2)	13.858 (57,9)		10.094 (42,1)	38.315 (11,0)
Não	107.113 (52,5)	96.946 (47,5)	58.907 (55,5)		47.289 (44,5)	310.255 (89,0)
Padrasto (n=347.474; 86,6%)						
Sim	5.171 (65,6)	2.708 (34,4)	9.137 (71,4)		3.657 (28,6)	20.673 (5,9)
Não	111.087 (52,9)	98.878 (47,1)	63.441 (54,3)		53.395 (45,7)	326,801 (94,1)
Desconhecido (n=348.176; 86,8%)						
Sim	4.236 (60,3)	2.789 (39,7)	3.345 (55,9)		2.641 (44,1)	13.011 (3,7)
Não	112.172 (53,0)	99.363 (47,0)	69.110 (55,9)		54.520 (44,1)	335.165 (96,3)
Irmão (n=348.305; 86,9%)						
Sim	2.251 (59,5)	1.534 (40,5)	2.944 (59,0)		2.050 (41,0)	8.779 (2,5)
Não	114.337 (53,2)	100.493 (46,8)	69.611 (55,8)		55.085 (44,2)	339.526 (97,5)
Cuidador (n=347.383; 86,6%)						
Sim	2.134 (59,4)	1.460 (40,6)	839 (55,2)		682 (44,8)	5.115 (1,5)
Não	114.049 (53,2)	100.316 (46,8)	71.544 (55,9)		56.359 (44,1)	342.268 (98,5)
Madrasta (n=347.617; 86,7%)						
Sim	590 (53,2)	519 (46,8)	878 (52,9)		782 (47,1)	2.769 (0,8)
Não	115.769 (53,4)	101.162 (46,6)	71.643 (56,0)		56.274 (44,0)	344.848 (99,2)
**Suspeita de uso de álcool** (n=217.387; 54,2%)						
Sim	13.019 (57,1)	9.773 (42,9)	10.022 (59,4)		6.845 (40,6)	39.659 (18,2)
Não	57.836 (52,3)	52.838 (47,7)	36.174 (53,9)		30.880 (46,1)	177.728 (81,8)

^a^% de completude: O percentual de completude foi calculado dividindo-se o número de registros existentes para dada variável pelo total de registros de notificações de violência infantil no período analisado.

As maiores taxas de notificação de violência infantil foram de negligência/abandono no grupo etário de 0-4 anos em ambos os sexos, com crescimento de 2011 a 2019 e padrão semelhante de redução entre 2020 e 2021. As taxas de violência sexual foram as segundas maiores para meninas de 0-4 anos e as maiores para aquelas de 5-9, com aumento em 2018 e 2019 e redução em 2020 e 2021. Para meninos, as taxas de notificação de violência física, psicológica e sexual apresentaram pouca variação ao longo do período. Entre meninas, as variações nas taxas de violência física e psicológica foram menores nos dois grupos etários e, para meninas de 5-9 anos, as taxas de negligência também foram muito semelhantes ao longo de todo o período (Figura 1).

**Figura 1:** Taxas nuais de notificações (por 100 mil crianças) de violência infantil física, psicológica, sexual e negligência/abandono entre crianças do sexo masculino de 0-4 anos (A), do sexo masculino de 5-9 anos (B), do sexo feminino de 0-4 anos (C) e do sexo feminino de 5-9 anos (D). Brasil, 2011 a 2021 (n=401.058)

As razões das taxas entre meninas e meninos revelaram que as violências em meninas apresentaram maiores taxas de notificação para violência sexual e psicológica em ambas as idades para todos os anos. Para violência sexual, entre crianças de 0-4 anos, as taxas de notificação foram de 3,33 vezes a 4,25 vezes maiores para as meninas em comparação aos meninos. Entre meninas de 5-9 anos, essa razão variou de 2,35 vezes a 3,84 vezes. As taxas de notificação de violência psicológica entre meninas variaram pouco entre as idades, sendo de 1,39 vez a 2,09 vezes maiores do que as dos meninos. Em relação à negligência, as taxas foram cerca de 10% menores para as meninas. Para violência física, as taxas entre meninas de 0-4 anos foram de 8% a 32% maiores no período quando comparadas às taxas dos meninos do mesmo grupo etário. Já entre o grupo de 5-9 anos, as taxas de violência física entre meninos e meninas praticamente não apresentaram diferença, mas mostraram uma leve tendência a serem menores entre as meninas (Tabela 3). 

**Tabela 3:** Razão das taxas de notificação de violência física, psicológica, sexual e negligência/abandono entre vítimas do sexo feminino e do masculino de 0-4 anos e de 5-9 anos de idade. Brasil, 2011 a 2021 (n=401.058)

Razão: taxas de violência no sexo feminino/ taxas de violência no sexo masculino para crianças de 0-4 anos de idade				
Ano	Física	Psicológica	Negligência	Sexual
2011	1,08	1,85	0,93	3,33
2012	0,95	1,80	0,89	3,36
2013	1,10	1,69	0,87	3,44
2014	1,13	1,66	0,91	3,62
2015	1,10	1,61	0,93	3,90
2016	1,17	1,60	0,91	3,35
2017	1,20	1,64	0,90	3,82
2018	1,28	1,74	0,92	4,04
2019	1,21	1,76	0,90	4,08
2020	1,32	2,09	0,89	4,33
2021	1,27	1,85	0,90	4,25
Razão: taxas de violência no sexo feminino/ taxas de violência no sexo masculino para crianças de 5-9 anos de idade				
2011	0,94	1,68	0,86	2,45
2012	0,91	1,60	0,81	2,35
2013	0,92	1,50	0,83	2,59
2014	0,89	1,39	0,79	2,51
2015	0,91	1,44	0,80	2,74
2016	0,99	1,52	0,88	2,82
2017	0,93	1,51	0,83	2,82
2018	0,94	1,47	0,84	2,94
2019	0,96	1,49	0,83	3,02
2020	1,07	1,77	0,85	3,41
2021	1,13	1,71	0,91	3,84

Na comparação entre faixas etárias, as maiores taxas de violência física e de negligência foram observadas entre crianças de 0-4 anos, em comparação àquelas de 5-9 anos. Essa diferença foi mais pronunciada para negligência, cujas taxas foram mais de 2,8 vezes maiores entre crianças de 0-4 anos em todos os anos. Por outro lado, as taxas de notificação de violência psicológica e sexual foram mais altas entre crianças de 5-9 anos (Tabela 4).

**Tabela 4:** Razão das taxas de notificação de violência física, psicológica, sexual e negligência/abandono entre vítimas de 0-4 anos e de 5-9 anos de idade do sexo feminino e do sexo masculino. Brasil, 2011 a 2021 (n=401.058)

Razão: taxas de violência 0-4 anos/ taxas de violência 5-9 anos para crianças do sexo feminino				
Ano	Física	Psicológica	Negligência	Sexual
2011	1,36	0,75	3,48	0,83
2012	1,29	0,69	3,21	0,87
2013	1,38	0,74	3,36	0,84
2014	1,50	0,80	3,33	0,87
2015	1,35	0,74	3,25	0,84
2016	1,34	0,74	3,25	0,75
2017	1,51	0,87	3,53	0,85
2018	1,42	0,79	3,44	0,87
2019	1,20	0,69	3,32	0,87
2020	1,51	0,77	3,67	0,89
2021	1,40	0,77	3,57	0,86
Razão: taxas de violência 0-4 anos/ taxas de violência 5-9 anos para crianças do sexo masculino				
2011	1,19	0,68	3,21	0,61
2012	1,25	0,62	2,95	0,61
2013	1,15	0,65	3,21	0,63
2014	1,18	0,67	2,87	0,60
2015	1,11	0,66	2,80	0,59
2016	1,12	0,70	3,13	0,63
2017	1,17	0,79	3,25	0,63
2018	1,04	0,66	3,17	0,63
2019	0,95	0,58	3,07	0,64
2020	1,22	0,66	3,48	0,70
2021	1,25	0,71	3,62	0,78

## Discussão

Esse estudo mostra alta proporção e taxas de notificação de violência para negligência, especialmente no grupo de 0-4 anos e de violência sexual, destacadamente para as meninas. A violência doméstica se revela como um problema grave, com maior proporção dos casos ocorrendo na residência da criança, tendo principalmente a mãe, mas também o pai como principais agressores.

As limitações do estudo residem na fonte de dados. Embora o Sinan seja uma ferramenta importante para a vigilância epidemiológica no Brasil, seus dados dependem do registro realizado pelos profissionais de saúde e pelas unidades, e a qualidade desses dados pode ser comprometida por erros de preenchimento e por omissão de notificações, o que pode resultar em subnotificação ou relatórios com importantes informações ausentes. Essa situação pode ser agravada em áreas com menor cobertura de serviços de saúde ou em populações mais vulneráveis. Outra limitação refere-se à duplicidade e à completitude das variáveis, o que limita a caracterização das ocorrências. Por exemplo, neste estudo, não foi possível utilizar a variável escolaridade devido à baixa completitude. Contudo, a não duplicidade foi observada para 99,9% dos registros, e, em geral, as variáveis analisadas apresentaram completitude acima de 85%, sendo valores acima do que é considerado aceitável para não duplicidade (>95%) e completitude boa (≥75,1%) (18). Algumas variáveis apresentaram completitude regular (de 50,1% a 75,0%): suspeita de uso de álcool e reincidência da ocorrência. Essa completitude baixa pode introduzir um viés, pois as análises baseadas em dados incompletos podem não representar todos os casos notificados. A validade externa do estudo é limitada, pois os dados não representam todos os casos de notificação em crianças brasileiras. 

A residência como principal local de ocorrência (74,8%) é consistente com outros estudos que analisaram dados do Sinan e com outras fontes de dados de violência no Brasil e no exterior (6,7,13). No entanto, alguns estudos locais apresentaram proporções menores, variando entre 50% e 60% dos casos de violência ocorridos na residência (14), o que sugere a existência de diferenças regionais e destaca a importância de compreender essas variações para adaptar as ações e as estratégias. A proximidade dos agressores, muitas vezes os pais, também explica a predominância da residência como local de ocorrência da violência (6).

As crianças pardas foram as com maior proporção de notificação. Quando consideradas as crianças negras (pardas e pretas), essa proporção atingiu 53%. Estudos nacionais (14) e internacionais (13,19) corroboram essa tendência, reconhecida também pela OMS (19). No entanto, alguns estudos brasileiros, incluindo-se análises regionais de dados do Sinan, indicam maior proporção de notificações entre crianças brancas (6). Dado que crianças negras representam cerca de 56,7% da população infantil brasileira (16), essa discrepância entre a proporção de crianças negras na população nacional e nas notificações de violência pode sugerir possíveis casos de subidentificação e/ou subnotificação de violências envolvendo crianças negras. Outra hipótese é o menor acesso dessas crianças aos serviços de saúde, o que poderia impactar na identificação e nas notificações. É importante refletir ainda se, devido ao racismo estrutural, os profissionais de saúde podem estar invisibilizando casos de violência contra crianças negras. Portanto, para que as medidas de prevenção e de controle da violência infantil sejam eficazes, é fundamental que sejam complementadas por políticas abrangentes de enfrentamento ao racismo estrutural.

Este estudo identificou uma alta proporção de agressores possivelmente sob efeito de álcool, alinhando-se à evidência da OMS de que crianças em famílias ou comunidades com histórico de abuso de álcool são mais vulneráveis à violência (20,21). Políticas para regular o uso de álcool, especialmente na presença de crianças, são essenciais. Recomenda-se que a ficha de notificação do Sinan inclua informações sobre o uso de outras substâncias.

Crianças de 0-4 anos apresentaram maiores taxas de notificação de negligência. Essa faixa etária é identificada como vulnerável à negligência devido à dependência delas aos cuidadores e à dificuldade em estabelecer contatos sociais protetores. Esses achados alinham-se com estudos que demonstram que a negligência é o tipo mais frequente de violência infantil, especialmente entre as mais novas (6,13,14,22). Estudos mostram que a negligência pode causar sérios danos ao desenvolvimento da criança, afetando sua cognição, atenção e comportamento social. Esses danos podem persistir ao longo da vida (22). Medidas preventivas universais são essenciais, tanto em nível individual quanto comunitário, e os cuidados de saúde desempenham um papel fundamental na detecção precoce da negligência (11,23). 

Situações de vulnerabilidade, como falta de acesso aos serviços de saúde e de recursos financeiros, muitas vezes resultam na desconsideração da negligência pelos profissionais, situação agravada em contextos de desigualdades sociais. Nesses casos, é importante reconhecer que a negligência não é apenas causada pelos pais ou de responsabilidade deles, mas também um reflexo das deficiências estruturais do Estado. É crucial que os profissionais identifiquem esses casos, os reconheçam como negligência e os notifiquem, mesmo quando não se trate de negligência parental. O registro diferencial entre negligência parental e estrutural pelo Sinan poderia favorecer a descrição mais fiel da situação desse tipo de violência, favorecendo estratégias adequadas de intervenção. A melhoria na qualidade dos registros também passa pela formação profissional, pois estudos apontam dificuldades dos profissionais em identificar e em abordar a negligência (23,24). Outro ponto importante que pode influenciar na decisão do profissional de não notificar casos suspeitos é a percepção distorcida de que a negligência seria uma forma de abuso com menor potencial para causar danos (24).

As taxas de notificação de violência sexual em meninas foram mais do que o dobro, chegando a mais de quatro vezes maiores do que as observadas para meninos. Os achados indicam um aumento do risco da violência sexual à medida que as meninas envelhecem (25). A cultura patriarcal e as relações de poder desempenham um papel crucial na perpetuação da violência sexual infantil, refletindo as desigualdades de gênero arraigadas na sociedade (26,27). Historicamente, as mulheres foram consideradas socialmente frágeis, submissas aos pais e aos companheiros, perpetuando-se um ciclo de dominação (26). Isso se reflete na subordinação das crianças, especialmente as meninas, que enfrentam maior risco de violência física e sexual devido à cultura do estupro e ao patriarcado (26). Combater o abuso sexual infantil envolve confrontar o machismo, a misoginia e a opressão de gênero.

A violência física foi o terceiro tipo mais notificado, diferente de outros estudos que reportaram maior proporção de violência física comparada ao abuso sexual (6,13). Isso pode sugerir uma possível normalização cultural da violência física infantil no Brasil, evidenciada pela aceitação do castigo físico como método disciplinar, apesar das iniciativas legais (Lei nº 13.010/2014) e debates recentes sobre o assunto (28,29). Essa aceitação pode se estender aos profissionais de saúde. Além disso, há uma crença normalizada, inclusive entre os profissionais, de que o abuso físico só deve ser considerado quando há lesões visíveis, o que pode resultar na não identificação de casos em que a criança não apresente sinais físicos e na subnotificação de casos (30).

O aumento das taxas de notificação de violência infantil ao longo dos anos observado neste estudo pode ser atribuído à implementação da Portaria n° 104/2011, que tornou a violência infantil um agravo de notificação compulsória em todos os níveis de atenção à saúde no Brasil. No entanto, a partir de 2020, observou-se uma diminuição nas taxas de notificação, possivelmente devido à pandemia de covid-19, o que pode ter levado à subnotificação. O isolamento social, o fechamento de escolas e a concentração dos serviços de saúde no enfrentamento da pandemia reduziram o contato das crianças com profissionais de saúde e a improbabilidade de estabelecerem laços sociais protetores no dia a dia. Mas essa diminuição nas notificações não deve ser interpretada como uma redução da ocorrência de violência, pois as incidências de violência aumentaram nesse período, corroborando a ideia de que a violência ocorre, principalmente, dentro do ambiente doméstico (19).

No Brasil, existem outros canais de notificação de violência infantil além do Sinan, como o Disque 100, mas os dados gerados não são integrados em um único sistema. O monitoramento dos casos ganharia muito com a integração, alinhando-se às orientações de organizações internacionais, como a OMS, que destacam a importância de sistemas de informações abrangentes e integrados para fortalecer a vigilância e o enfrentamento da violência. Além disso, é fundamental esclarecer os profissionais de saúde sobre a compulsoriedade da notificação e o uso do Sinan como sistema de informação padrão, pois evidências revelam o desconhecimento (30). 

Apesar das políticas existentes de combate à violência infantil no Brasil, é evidente a necessidade de maior investimento em outras políticas para reduzir essas taxas, destacando-se a importância de políticas de gênero, de respeito e de proteção do sexo feminino, de cultura da paz, de combate ao racismo, além da integração da educação sexual desde cedo. Ademais, é crucial capacitar melhor os profissionais de saúde e de educação para lidar com o problema. Destaca-se que a negligência e a residência são os pontos principais de ocorrência de violência infantil registrada no Brasil no período estudado, o que ressalta o papel crucial da atenção primária à saúde. Reforçar a vigilância sobre as violências infantis, a integrando à atenção primária, pode ser uma medida eficaz no enfrentamento da violência infantil.
